# Preoperative chemoradiotherapy using tegafur/uracil, oral leucovorin, and irinotecan (TEGAFIRI) followed by oxaliplatin-based chemotherapy as total neoadjuvant therapy for locally advanced rectal cancer: the study protocol for a phase II trial

**DOI:** 10.1186/s12885-023-10941-z

**Published:** 2023-05-17

**Authors:** Shinya Abe, Kazushige Kawai, Hiroaki Nozawa, Kazuhito Sasaki, Koji Murono, Shigenobu Emoto, Yuichiro Yokoyama, Hiroyuki Matsuzaki, Yuzo Nagai, Yuichiro Yoshioka, Takahide Shinagawa, Hirofumi Sonoda, Yoko Yamamoto, Koji Oba, Soichiro Ishihara

**Affiliations:** 1 Department of Surgical Oncology, Graduate School of Medicine, University of Tokyo, 7-3-1 Hongo, Bunkyo-ku, Tokyo, 113-0033 Japan; 2 Department of Surgery, Tokyo Metropolitan Cancer and Infectious Diseases Center Komagome Hospital, Bunkyo-ku, Tokyo, Japan; 3 Department of Biostatistics, Graduate School of Medicine, The University of Tokyo, Bunkyo-ku, Tokyo, Japan

**Keywords:** Rectal cancer, Radiotherapy, Chemotherapy, Total neoadjuvant therapy

## Abstract

**Background:**

Total neoadjuvant therapy (TNT) is a novel treatment strategy that is an alternative to preoperative chemoradiotherapy (CRT) for locally advanced rectal cancer (LARC). However, an optimal protocol for TNT has not yet been established. The present study will be an open-label, single-arm, single-center trial to develop a new protocol.

**Methods:**

Thirty LARC patients at high risk of distant metastasis will receive CRT consisting of long-course radiation, concurrent with tegafur/uracil, oral leucovorin, irinotecan (TEGAFIRI), followed by mFOLFOX-6 or CAPOX before undergoing surgery.

**Discussion:**

Since previous findings showed a high percentage of grade 3–4 adverse events with the TEGAFIRI regimen for CRT and TNT, the primary outcome of this study will be safety and feasibility. Our regimen for CRT consists of the biweekly administration of irinotecan for good patient compliance. The novel combination approach of this treatment may improve the long-term outcomes of LARC.

**Trial Registration:**

Japan Registry of Clinical Trials jRCTs031210660.

**Supplementary Information:**

The online version contains supplementary material available at 10.1186/s12885-023-10941-z.

## Introduction

A new approach for locally advanced rectal cancer (LARC), such as Stage II/III lower rectal cancer, is preoperative chemoradiotherapy (CRT) followed by total mesorectal excision (TME) and adjuvant chemotherapy. Preoperative CRT has reduced local recurrence [1, 2] and improved long-term outcomes in patients who achieved pathological complete remission (pCR) [3]; however, distant relapse occurs in the majority of LARC patients receiving CRT [4]. Although adjuvant chemotherapy also plays an essential role in systemic recurrence in LARC patients receiving CRT [5], adherence to adjuvant chemotherapy with FOLOX and CAPOX is poor after surgery for a number of reasons, including postoperative complications, disease progression, and the toxic effects of pre- and postoperative treatments [2, 6]. Furthermore, in cases receiving long-course radiotherapy (RT), the interval between the diagnosis of LARC and systemic chemotherapy as postoperative treatment ranges between approximately four and six months. Systemic metastasis has been detected in this period in some cases. Total neoadjuvant chemotherapy (TNT) is now being performed to increase compliance with postoperative treatments and start systemic chemotherapy earlier for micrometastasis. The concept of TNT for LARC was initially examined in a clinical trial by Chau et al., who reported that induction or consolidation therapy achieved pCR in more than 20% patients receiving neoadjuvant capecitabine/oxaliplatin [7, 8]. Randomized control trials on TNT have recently been performed, in which induction or consolidation therapy or long- or short-course RT were selected [9–11]. The randomized RAPIDO trial, which used TNT consisting of short-course CRT and consolidation chemotherapy with the oxaliplatin regimen, reduced the probability of distant metastasis, but did not prolong overall survival (OS) or achieve better local control than long-course CRT [9]. The randomized PRODIGE 23 trial treated patients with neoadjuvant therapy that included FOLFIRINOX and a long-course CRT protocol, and reported longer disease-free survival (DFS); however, it did not stratify the OS curve compared to long-course CRT [10]. Based on the findings of these two trials, neoadjuvant systemic chemotherapy with the oxaliplatin regimen may reduce distant metastasis. Furthermore, a non-randomized phase II trial demonstrated that long-course CRT and two or four cycles of mFOLFOX-6 prolonged DFS [12]. Therefore, we selected long-course CRT followed by more than two cycles of the oxaliplatin regimen in the present trial on LARC patients at a high risk of distant metastasis and whose features included a suspected T4b tumor, lateral pelvic lymph node metastasis, or extramural venous invasion [9]. We also developed long-course CRT accompanied by oral 5-FU drugs with irinotecan (TEGAFIRI) in a clinical phase I/II study to increase the rate of pCR [13]. In this clinical trial, we aim to develop a new protocol that is consistent with the TEGAFIRI regimen for CRT and the oxaliplatin-based regimen for consolidation chemotherapy for LARC.

## Materials and methods

### Study design

**Fig. 1:**
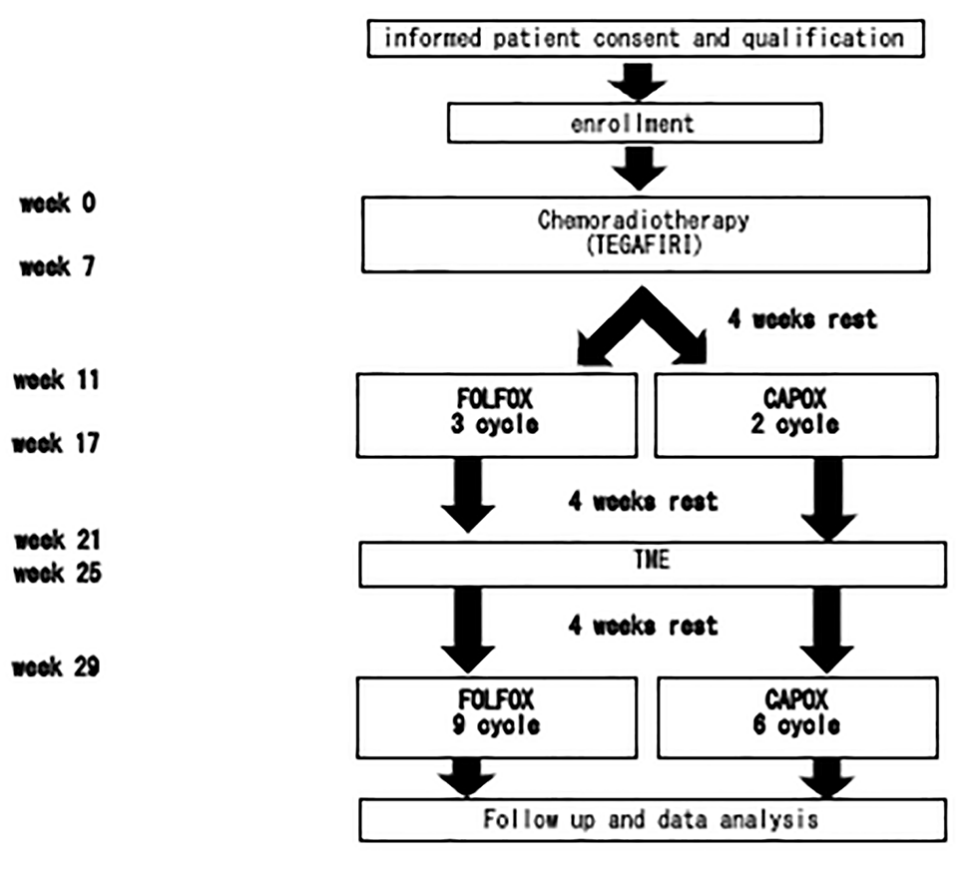
Flow diagram of the trial

The present study will be an open-label, single-arm, single-center prospective phase-II trial to establish the total neoadjuvant chemotherapy protocol. Preoperative CRT, followed by three cycles of mFOLFOX-6 chemotherapy or two cycles of CAPOX chemotherapy will be performed prior to TME (open, laparoscopic, or robotic). Nine cycles of mFOLFOX-6 chemotherapy or six cycles of CAPOX chemotherapy will then be administered postoperatively to patients with resectable locally advanced rectal adenocarcinoma at a high risk of distant metastasis, including the pretreatment status of a suspected T4b tumor, lateral pelvic lymph node metastasis, or extramural venous invasion. The treatment algorithm is shown in Fig. 1. Pretreatment CT and MRI will be used to diagnose all patients. The study protocol has been approved by the Ethics Committee of the University of Tokyo Hospital and the University of Tokyo Clinical Research Ethical Committee (approval No.: 2021508SP) and is registered in the Japan Registry of Clinical Trials (jRCT, approval No.: jRCTs031210660). This study was opened in March 2022. In modification protocol, including explanatory documents and consent forms, and various procedure manuals are to be changed, the principal investigator must consult with the University of Tokyo clinical research review committee prior to the change. After receiving the board’s review and approval, the change’s details will be reported to the hospital director. Then, submit a notification of changes to the implementation plan to the Minister of Health, Labor and Welfare by registering with jRCT.

### Sample size

The target sample size was set as 30 patients based on the confidence interval (CI) width. According to a previous clinical trial [9], we estimated the incidence of adverse events (AE) to be 40% in the present study protocol. The two-sided 95% CI ranged between 22.7 and 59.4% with the enrollment of 30 patients. A previous study showed that the incidence of AEs in the historical control was approximately 30% [13]; therefore, 30 patients fulfilled the desired precision because the incidence of AEs in the estimated upper 95% CI is not more than twice that in the control group.

### Study objectives

The primary outcome will be the incidence of AEs during total neoadjuvant therapy at the end of adjuvant FOLFOX or CAPOX therapy. AEs will be evaluated according to the National Cancer Institute Common Terminology Criteria for Adverse Events (CTCAE) version 5.0. Secondary objectives will be the evaluation of long-term outcomes, including distant relapse-free survival at 3 years, relapse-free survival at 3 years, the local recurrence rate at 3 years, and overall survival. Furthermore, pCR rates; postoperative morbidity and mortality rates; the dose intensity for each regimen, including CRT, neoadjuvant chemotherapy (NAC), and adjuvant therapy; and compliance with the study protocol, defined as completing full-dose treatment in each regimen, will be secondary endpoints.

### Patient selection

Thirty patients with locally advanced rectal adenocarcinoma will be enrolled. Patients will be 20–80 years old and meet inclusion and exclusion criteria (Table 1).

**Table 1:** Inclusion and exclusion criteria

Inclusion Criteria			Exclusion Criteria	
1)	A tumor located below the peritoneal reflection or a rectal tumor reaching the peritoneal reflection		1)	Patients with a history of drug hypersensitivity
2)	Patients with histologically confirmed adenocarcinoma		2)	Patients for whom UFT/UZEL is contraindicated
3)	Patients judged to fall under any of the following by a preoperative diagnosis using MRI or CT		3)	Patients for whom the administration of CPT-11 is contraindicated
	(1) Infiltration into the surrounding organs (T4b, any N)		4)	Patients with UGT1A1*6/*6, UGT1A1*28/*28, and heterozygotes (UGT1A1*6/*28)
	(2) Suspected advanced lymph node metastasis, such as lateral lymph node metastasis		5)	Patients for whom the administration of 5-FU is contraindicated
	(3) Suspected extramural venous invasion		6)	Patients for whom the administration of LV is contraindicated
4)	Patients without distant organ metastasis		7)	Patients for whom the administration of L-OHP is contraindicated
5)	Patients aged between 20 and 80 years (at the time of obtaining consent)		8)	Patients for whom the administration of capecitabine is contraindicated
6)	Patients in whom radiation therapy, chemotherapy, or hormone therapy has not been performed as a pretreatment		9)	Patients receiving flucytosine, phenytoin, and potassium warfarin
7)	Patients who ingest food and may be orally administered drugs		10)	Patients with active infectious diseases (fever 38.0 °C or higher)
8)	Patients in whom the functions of the major organs are sufficiently maintained.		11)	Patients with serious complications
	The doctor will make a judgment based on the following values:		12)	Patients with diarrhea (more than 4 times a day or watery stools)
	a. White blood cell count: 4,000/mm^3^ and higher, and 12,000/mm^3^ and lower		13)	Patients with clinical brain metastases and those with a history of brain metastases
	b. Number of neutrophils: 2,000/mm^3^ or higher			Patients with simultaneous double cancer or metachronous double cancer with a disease-free period of 5 years or less
	c. Platelet count: 100,000/mm^3^ or higher		14)	Pregnant or lactating women or those with the desire to become pregnant
	d. Hemoglobin: 9.0 g/dl or higher		15)	Men who wish to have children
	e. Total bilirubin: 1.5 mg/dl or lower		16)	Other patients that the doctor in charge deems inappropriate as a target
	f. AST (GOT)/ALT (GPT): 100 IU/L and lower			
	g. Serum creatinine clearance using the Cockcroft-Gault estimation formula: 60.0 mL/min and more			
	* * If creatinine clearance by 24-hour urine collection is measured, that value is used.			
	If there is no measured value, calculate the estimated value using the Cockcroft-Gault estimation formula:			
	Male: creatinine clearance = body weight × (140-age) / (72 × serum creatinine level)			
	Female: creatinine clearance = 0.85 × {weight × (140-age) / (72 × serum creatinine level)}			
9)	Patients with no abnormal electrocardiogram findings (abnormalities without clinical issues are acceptable)			
10)	Patients with an Eastern Cooperative Oncology Group Performance Status of 0 to 1			
11)	Patients without sensory neuropathy			
12)	The research subject has given written consent to participate in the study			

### Data collection, monitoring, and auditing

Table 2 shows the schedule for the assessment. The principal investigator and sub-study investigator will monitor and assess the clinical course, safety, and efficacy outcomes recorded in medical records and complete the case report form (CRF). All CRF will be submitted to a separate department for data management, which will be appointed by the principal investigator, in the University of Tokyo. The monitor will perform verification based on “Written procedure for the study monitoring” during the study. Among CRFs, severe cases will be reported to the Ethical Committee within 30 days. No auditing is planned as part of this exploratory study.

**Table 2:** Assessment schedule summary

	Pretreatment assessment	Chemoradiotherapy (CRT) UFT/UZEL+CPT-11 + RT	Post CRT	Consolidation chemotherapy (FOLFOX or CAPOX)	Post CTx
Informed consent•Case registration	●				
Obtaining medical history	●				
Subjective and objective findings	●	●	●	●	●
Laboratory tests	●	●	●	●	●
Imaging test	●		●		●
Confirmation of medication status		●		●	

### Data analysis

Patient baseline characteristics will be used to calculate summary statistics and distributions. The frequency and proportions of the categories for nominal variables are displayed for each group. Summary statistics (the number of cases, mean, standard deviation, minimum, median, and maximum) for continuous variables will be calculated for each group. The primary outcome analysis as an interval estimation of the incidence of AEs (CTCAE ≥ grade 3) is performed using an accurate CI based on the binomial distribution. The incidence of severe adverse events (AEs), the primary endpoint, might be higher in the TNT group than in patients who received preoperative chemoradiation with UFT/UZEL plus CPT-11 at our department (historical control group). If necessary, the ratio of severe AEs will be compared using Fisher’s exact calculus test.

### Treatment plan

Patients who meet the eligibility criteria will be informed of all details about the study procedure. They will receive CRT, mFOLFOX-6, or CAPOX; undergo radical surgery at approximately week 21; and receive another course of mFOLFOX-6 or CAPOX (Fig. 1). Only patients who voluntarily participate in the study and provide informed consent will be enrolled.

### Preoperative CRT

CRT will be performed as previously described [13]. Briefly, CRT consists of long-course radiation (50.4 Gy/28 fractions, five days per week) on days 1–38, concurrent with oral and intravenous chemotherapy (TEGAFIRI; tegafur/uracil, oral leucovorin, irinotecan). During RT, UFT (300 mg/m^2^/day for less than 1.17 m [2], 400 mg/m^2^/day from 1.17 to 1.49 m [2], 500 mg/m^2^/day from 1.50 to 1.83 m [2], and 600 mg/m^2^/day for more than 1.83 m [2]) and LV 75 (mg/day) will be orally administered on the days of RT, while irinotecan (80 mg/m^2^) will be intravenously administered on days 1, 15, 29, and 43.

A physical examination, laboratory test, and AE assessment will be performed at every irinotecan injection. AEs that occur during or after the cycle administration will be collected.

### Neoadjuvant systemic chemotherapy

Four to eight weeks after the completion of RT, patients will receive NAC, including three cycles of mFOLFOX-6 or two cycles of CAPOX. The mFOLFOX-6 regimen consists of the following: oxaliplatin 85 mg/m^2^ intravenously administered and 5-FU 400 mg/m^2^ administered as an intravenous bolus on day 1, followed by 2400 mg/m^2^ intravenously administered over 24 h on days 1–2, and leucovorin 200 mg/m^2^ intravenously administered on day 1. One cycle of mFOLFOX-6 is 14 days. The CAPOX regimen consists of the following: oxaliplatin 130 mg/m^2^ intravenously administered on day 1 and capecitabine 2000 mg/m^2^ orally administered twice a day on days 1–14 in every cycle. One cycle of CAPOX is 21 days. A physical examination, laboratory test, and AE assessment will be performed at the time of every oxaliplatin injection. AEs that occur during or after the cycle administration will be collected.

### Rectal resection

Radical surgery will be performed 4 to 8 weeks after the completion of NAC. Lateral lymph node dissection will be selectively performed on patients with suspected lateral lymph node metastasis before CRT. All resected specimens will be pathologically analyzed according to the American Joint Committee on Cancer classification [14]. The pathological therapeutic response of the primary tumors will be assessed according to the Japanese Classification of Colorectal, Appendiceal, and Anal Carcinoma of the Japanese Society for Cancer of the Colon and Rectum [15]. The frequency and grade of surgical complications within 30 days of surgery will be evaluated using the Clavien-Dindo classification.

### Adjuvant systemic chemotherapy

Patients will receive adjuvant chemotherapy within 12 weeks of surgery, including nine cycles of mFOLFOX-6 or six cycles of CAPOX. AEs that occur during or after each cycle administration will be collected. A physical examination, laboratory test, and AE assessment will be performed at the time of every oxaliplatin injection. If patients cannot start to receive adjuvant chemotherapy or if adjuvant chemotherapy is discontinued prematurely, the follow-up period will be started in these patients.

#### Chemotherapy administration criteria and dose reduction criteria

Chemotherapy administration criteria are shown in Supplementary Table 1. The criteria consist of number of hematologic toxicities, including white blood cells, neutrophil, platelets, and non-hematologic toxicities, including nausea and hand-foot syndrome. Chemotherapy is interrupted until the criteria are met. The dose reduction criteria and the reduced doses are shown in Supplementary Tables 2 and Supplementary Tables 3, respectively. Each chemotherapeutic drugs should be reduced when hematologic and non-hematologic toxicity ≥ Grade 3 is observed. In addition, doses can be reduced at the physician’s discretion to ensure safety.

#### Rescue and prohibited concomitant medication

Concomitant use of aprepitant, fosaprepitant meglumine, and granisetron prevent adverse events during systemic chemotherapy. Palonosetron, steroid, and d-chlorpheniramine maleate could be combined with the oxaliplatin regimen (Day 1). Palonosetron and steroids could be used concomitantly during the administration of CPT-11 (Day 1). In addition, supportive care drugs for the several symptoms caused by chemotherapy are allowed to use under the consideration of investigators.

Hormonal therapy other than steroids and concomitant therapy such as immunotherapy is prohibited. Drugs or treatments with anti-tumor effects not listed in the protocol are also prohibited.

#### Adherence assessments

Multiple occupations, including investigators and pharmacists, will assess medication adherence at every follow-up visit or admission during pre- and postoperative chemoradiation and chemotherapy to enhance data validity. These data are recorded on the appropriate case report form.

### Follow-up

Postoperative surveillance is performed according to the Japanese Society for Cancer of the Colon and Rectum guidelines [16]. In this surveillance, patients will receive a physical examination, blood examination, including a complete blood count, routine chemistry, including liver and kidney function tests, and the measurement of carcinoembryonic antigen levels in serum every three months. In addition, an assessment by CT (every six months) and colonoscopy (every year) will be performed.

### Safety evaluation and reporting of adverse events

The principal and sub-study investigators will monitor and assess the clinical course, safety, and efficacy outcomes recorded in medical records. All adverse events during pre- and postoperative treatment and postoperative complications 30 days after surgery need to be reported in the case report form (CRF). Among CRFs, severe cases will be reported to the Ethical Committee within 30 days. Then investigators will review all events and reactions. The National health system covers indemnity for negligent harm in patients enrolled in the study. After completion or discontinuation of this clinical study, the participants will be performed necessary examinations and observations to ensure their safety.

## Discussion

TNT has recently been added as a treatment option for LARC in the National Comprehensive Cancer Network and European Society for Medical Oncology consensus guidelines [17, 18]. However, combination therapy with an optimal protocol for TNT that improves the long-term outcomes of LARC has yet to be established. Choices from the following four areas need to be selected: induction and consolidation therapies; the duration of RT (short or long course); the regimens of chemotherapy combined with RT; and the regimens of systemic chemotherapy for induction or consolidation therapy.

The control of local recurrence is essential for LARC. Long-course CRT followed by TME approximately 6 to 8 weeks after the completion of RT resulted in superior treatment effects to those after 2 weeks of RT [19]; therefore, consolidation therapy is an appropriate treatment that effectively utilizes the waiting period for TME. Although the RAPIDO trial did not compare short- and long-course RT in the TNT protocol, this trial did not indicate whether TNT was superior to conventional CRT for the control of local recurrence [9]. Therefore, we expect long-course CRT with consolidation therapy to improve both local and distant relapse-free survival.

To increase the treatment efficacy of CRT, oxaliplatin-based chemotherapy has been used in several clinical trials and achieved high pCR rates [20–24]. In Japan, the SHOGUN trial, which used CRT consisting of S-1 and oxaliplatin, reported a pCR rate of 27.3% [25, 26]. However, a meta-analysis of 10 randomized controlled trials demonstrated that the addition of oxaliplatin to fluorouracil CRT did not improve overall survival (OS), DFS, or local recurrence [27]. On the other hand, another meta-analysis demonstrated that the addition of oxaliplatin significantly decreased treatment failure due to distant metastasis, but did not improve OS and DFS [28]. These findings provide support for the preoperative administration of oxaliplatin to control distant metastasis.

We focused on irinotecan, another key anticancer drug for colorectal cancer. As a solid tumor grows, blood supply to the center of the tumor becomes insufficient, and, thus, the concentration of oxygen decreases. In an in vitro experiment, we demonstrated that hypoxia-inducible factor 1α (HIF-1α), which was induced in this hypoxic environment, prevented cell death and promoted the therapeutic resistance of colorectal cancer cells [29]. We also found that irradiation induced the expression of HIF-1α in colon cancer cells and hypoxia. Irinotecan is metabolized to SN-38 in the body, and SN-38 suppressed HIF-1α expression induced by irradiation and enhanced radiosensitivity [30]. Several phase II clinical trials on the concurrent use of irinotecan CRT reported higher pCR rates [31–36]. A recent multicenter, randomized, phase III trial showed significantly higher pCR rates of 30%; however, significantly higher grade 3–4 toxicities occurred in 38% of patients [37]. The weekly administration of irinotecan was selected in these clinical trials and induced severe AEs, particularly leukopenia, neutropenia, and diarrhea.

Our regimen for CRT consists of the biweekly administration of irinotecan for good patient compliance and a total irinotecan dose of 320 mg/m^2^, based on the total irinotecan dose of 240 mg/m^2^ recommended by Klautke et al. to achieve pCR [38].

Our primary endpoint will be the safety and feasibility of our protocol. Although we hope our protocol improves long-term outcomes, the concurrent administration of irinotecan with CRT and the previous TNT protocol induced high grade 3–4 AEs in 38% of patients in the RAPIDO trial and in 27% of those in the PRODIGE 23 trial [9, 10]. Since a TNT scheme has not yet been established, the RAPIDO trial comprises full-dose chemotherapy before surgery without the adjuvant setting [9], while the Stellar trial includes neoadjuvant and adjuvant chemotherapy settings [39]. Our treatment protocol comprised CRT concurrent with irinotecan and the continuous administration of oxaliplatin, which may increase the ratio of high-grade AEs during the preoperative treatment term. Therefore, we need to consider the cycle number of preoperative systemic chemotherapy, strictly manage patient conditions, and develop a feasible TNT protocol for LARC.

Furthermore, improvements to preoperative treatments for LARC, such as TNT, including our treatment strategy, may increase clinical and pathological CR rates. Although some patients with clinical CR desire non-operative management, difficulties are associated with predicting pCR without surgery due to a lack of criteria. Recent studies demonstrated that diffusion-weighted MRI helped to discriminate between pCR and no-pCR patients [40, 41]. Therefore, the results of imaging tests performed during this clinical study will also be available for the establishment of solid scientific evidence.

## Electronic supplementary material

Below is the link to the electronic supplementary material.

Supplementary Material 1

## Data Availability

Data supporting this study’s findings are written by hand on paper and stored in a secured server, so the data is not publicly available but restricted to the acknowledged study personnel as per study protocol. Data processing, from data collection to database lock, will be carried out following Good Clinical Practice. The Data Manager will track all CRFs received in the Data Management Unit. Data Monitoring Unit will check the consistency of data. In addition, the statistician will analyze the final data, prepare an analysis report, and then submit it to the principal investigator. After the final analysis for each evaluation item, after the approval of the University of Tokyo Clinical Research Review Committee, it is possible to present a paper at an academic conference.

## Declarations

### Biological samples collection

Blood samples and remnant tissue specimens taken at the surgery will be stored to analyze biomarkers related to treatment evaluation or disease in this study in the future.
