# Supplementary material for: Preoperative chemoradiotherapy using tegafur/uracil, oral leucovorin, and irinotecan (TEGAFIRI) followed by oxaliplatin-based chemotherapy as total neoadjuvant therapy for locally advanced rectal cancer: the study protocol for a phase II trial
Source: BMC Cancer. 2023 May 17;23:450. doi: 10.1186/s12885-023-10941-z (PMC10193805; doi:10.1186/s12885-023-10941-z)
Supplement: Supplementary file 1 — Supplementary Material 1 [file 12885_2023_10941_MOESM1_ESM.docx]

**Supplementary Table 1.** Criteria for the administration of chemotherapy

| CPT-11 |
| --- |
| 1. White blood cells ≥3,000/mm^3^ 2. Neutrophils ≥1,500/mm^3^ |
| 1. Platelets ≥100,000/mm^3^ |
| 1. T-Bilirubin ≤1.5-fold the standard upper limit value of JCCLS* |
| 1. AST ≤5-fold the standard upper limit value of JCCLS* 2. ALT ≤5-fold the standard upper limit value of JCCLS* |
| 1. Creatinine ≤1.2-fold the standard upper limit value of JCCLS* |
| 1. No symptom of active infections |
| 1. Nausea, vomiting, diarrhea, and hand-foot syndrome ≤Grade 1 |
| 1. No peripheral neuropathy |
| 1. Other symptoms ≤Grade 2 |

*Japanese Committee for Clinical Laboratory Standards

| UFT/UZEL |
| --- |
| 1. White blood cells ≥2,000/mm^3^ 2. Neutrophils ≥1,000/mm^3^ |
| 1. Platelets ≥100,000/mm^3^ |
| 1. T-Bilirubin ≤ 1.5-fold the standard upper limit value of JCCLS* |
| 1. AST ≤3-fold the standard upper limit value of JCCLS* 2. ALT ≤3-fold the standard upper limit value of JCCLS* |
| 1. Creatinine ≤1.2-fold the standard upper limit value of JCCLS* |
| 1. No symptom of active infections |
| 1. Nausea, vomiting, diarrhea, and hand-foot syndrome ≤Grade 1 |
| 1. No peripheral neuropathy |
| 1. Other symptoms ≤Grade 2 |

*Japanese Committee for Clinical Laboratory Standards

| FOLFOX regimen |
| --- |
| 1. White blood cells ≥3,000/mm^3^ 2. Neutrophils ≥1,500/mm^3^ |
| 1. Platelets ≥75,000/mm^3^ |
| 1. T-Bilirubin ≤1.5-fold the standard upper limit value of JCCLS* |
| 1. AST ≤3-fold the standard upper limit value of JCCLS* 2. ALT ≤3-fold the standard upper limit value of JCCLS* |
| 1. Creatinine ≤1.2-fold the standard upper limit value of JCCLS* |
| 1. No symptom of active infections |
| 1. Nausea, vomiting, diarrhea, and hand-foot syndrome ≤ Grade 1 |
| 1. Peripheral neuropathy ≤Grade 2 |
| 1. Other symptoms ≤Grade 2 |

*Japanese Committee for Clinical Laboratory Standards

| CAPOX regimen |
| --- |
| 1. White blood cells ≥3,000/mm^3^ 2. Neutrophils ≥1,500/mm^3^ |
| 1. Platelets ≥75,000/mm^3^ |
| 1. T-Bilirubin ≤1.5-fold the standard upper limit value of JCCLS* |
| 1. AST ≤3-fold the standard upper limit value of JCCLS* 2. ALT ≤3-fold the standard upper limit value of JCCLS* |
| 1. Creatinine ≤1.2-fold the standard upper limit value of JCCLS* |
| 1. No symptom of active infections |
| 1. Nausea, vomiting, diarrhea, and hand-foot syndrome ≤ Grade 1 |
| 1. Peripheral neuropathy ≤Grade 2 |
| 1. Other symptoms ≤Grade 2 |

*Japanese Committee for Clinical Laboratory Standards

**Supplementary Table 2.** Criteria for dose reductions

|  | CPT-11 | UFT/UZEL | FOLFOX |
| --- | --- | --- | --- |
| White blood cells <2,000/mm^3^ | reduction | reduction | no change |
| Neutrophils <1,500/mm^3^ | no change | no change | reduction |
| Neutrophils <1,000/mm^3^ | reduction | reduction | no change |
| Platelets <75,000/mm^3^ | reduction | reduction | reduction |
| T-Bilirubin ≥2.5 mg/dL | reduction | reduction | reduction |
| Stomatitis ≥Grade 3 | no change | reduction | reduction |
| Non-hematological toxicity, excluding nausea, vomiting, anorexia, and hair loss ≥Grade 3 | reduction | reduction | reduction |
| Adverse events requiring  postponement for more than 15 days | reduction | no change | no change |

| CAPOX regimen |  | Number | Capecitabine | oxaliplatin |
| --- | --- | --- | --- | --- |
| Non-hematological toxicity | Grade 3 | 1 | 1^st^ reduction | 1^st^ reduction |
|  |  | 2 | 2^nd^ reduction | 2^nd^ reduction |
|  | Grade 4 | 1 | 2^nd^ reduction | 2^nd^ reduction |
| Hematologic toxicity | Grade 2 | 1 | no change | no change |
|  |  | 2  3 | 1^st^ reduction | no change |
|  |  |  | 2^nd^ reduction | no change |
|  | Grade 3 | 1 | 1^st^ reduction | no change |
|  |  | 2 | 2^nd^ reduction | 1^st^ reduction |
|  | Grade 4 | 1 | 2^nd^ reduction | 2^nd^ reduction |

**Supplementary Table 3.** Reduced doses

UFT/UZEL

| Body surface area | Initial dose | | Reduction 1 | | Reduction 2 | |
| --- | --- | --- | --- | --- | --- | --- |
|  | UFT mg/day | UZEL mg/day | UFT mg/day | UZEL mg/day | UFT mg/day | UZEL mg/day |
| <1.17 m^2^ | 300 | 75 | 0 | 0 | 0 | 0 |
| 1.17 m^2^ ≤BSA <1.49m^2^ | 400 | 75 | 300 | 75 | 0 | 0 |
| 1.49 m^2^ ≤BSA <1.83 m^2^ | 500 | 75 | 400 | 75 | 300 | 75 |
| ≥1.83 m^2^ | 600 | 75 | 500 | 75 | 400 | 75 |

| FOLFOX regimen | Initial dose | Reduction 1 | Reduction 2 |
| --- | --- | --- | --- |
| 5-FU bolus | 400 mg/m^2^ | 200 mg/m^2^ | 0 mg/m^2^ |
| 5-FU continuous | 2,400 mg/m^2^ | 2,000 mg/m^2^ | 1,600 mg/m^2^ |
| oxaliplatin | 85 mg/m^2^ | 65 mg/m^2^ | 50 mg/m^2^ |

| CAPOX regimen | Initial dose | Reduction 1 | Reduction 2 |
| --- | --- | --- | --- |
| oxaliplatin | 130 mg/m^2^ | 100 mg/m^2^ | 85 mg/m^2^ |

Capecitabine

| Body surface area | Initial dose | Reduction 1 | Reduction 2 |
| --- | --- | --- | --- |
| <1.36 m^2^ | 2,400 mg/m^2^/day | 1,800 mg/m^2^/day | 1,200 mg/m^2^/day |
| 1.36 m^2^ ≤BSA <1.66m^2^ | 3,000 mg/m^2^/day | 2,400 mg/m^2^/day | 1,800 mg/m^2^/day |
| 1.66 m^2^ ≤BSA <1.96 m^2^ | 3,600 mg/m^2^/day | 3,000 mg/m^2^/day | 2,400 mg/m^2^/day |
| ≥1.96 m^2^ | 4,200 mg/m^2^/day | 3,600 mg/m^2^/day | 3,000 mg/m^2^/day |
